# Declining maerl vitality and habitat complexity across a dredging gradient: Insights from *in situ* sediment profile imagery (SPI)

**DOI:** 10.1038/s41598-019-52586-8

**Published:** 2019-11-11

**Authors:** Guillaume Bernard, Alicia Romero-Ramirez, Adeline Tauran, Michael Pantalos, Bruno Deflandre, Jacques Grall, Antoine Grémare

**Affiliations:** 1 CNRS, EPOC, UMR 5805, F-33615, Pessac, France; 2 Université de Bordeaux, EPOC, UMR 5805, F-33615, Pessac, France; 3 IUEM UMS 3113, UBO, Brest, France

**Keywords:** Ecology, Ecology, Environmental sciences, Limnology, Ocean sciences

## Abstract

Maerl beds form complex biogenic benthic habitats, characterized by high productivity as well as diverse biological communities. Disturbances associated with extraction and/or fishing activities using mobile bottom-contacting gears such as clam-dredges induce the most severe and long-term effects on these fragile habitats. We here investigated the effects of dredge-fishing on maerl in the bay of Brest (France). We quantified maerl beds structure and vitality across a fine scale quantified dredging intensity gradient through the acquisition of *in-situ* images of beds cross-section using Sediment Profile Imaging system (SPI). Declines in the proxies of maerl vitality and habitat complexity were measured across the gradient, and were associated with significant changes in the vertical distribution of live and dead maerl as well as of interstitial space. Fishing with dredges caused maerl mortality, substratum compaction, and decreasing habitat complexity. SPI imaging techniques also allowed for an assessment of changes in spatial heterogeneity that dredging created on several aspects of the structure and vitality of maerl beds. It suggests that direct and indirect disturbances induced by dredging are not acting at the same spatial scale, and can thereby differentially affect the ecosystem functions linked to vitality and habitat complexity.

## Introduction

Maerl beds refer to free-living (or unattached) non-geniculate corralline algae (Corallinophycidae, Rhodophyta), also called rhodoliths, accumulating on soft sediment seafloor to form highly 3D complex structured biogenic benthic habitats^1–3^. The structural complexity of this habitat noticeably involves the creation of numerous ecological niches for fauna and flora within interstitial spaces between algal branches^4,5^. Maerl beds thus constitute “biodiversity hotspots” hosting highly diverse and abundant benthic fauna communities characterized by a mixture of hard- and soft-substrata species^5–8^ as well as rich algal communities^9^. They also constitute nursery areas for several fish and shellfish exploited species^6,10^. Maerl beds are highly productive habitats that can be compared to seagrass meadows in term of contribution to overall ecosystem functioning in temperate waters^11^. In particular, it has been suggested that they play a major role in biogeochemical cycles as significant carbon and nitrogen sinks^3,12–14^.

Maerl beds worldwide are facing several threats that would in the long-run affect their functioning and survival^15^. These include global warming and ocean acidification hindering the calcium carbonate formation by the coralline algae^16,17^, aquaculture, invasive species, or eutrophication that limit photosynthesis through a reduction of light availability at the seafloor^18^. Beside these, physical disturbances associated with extraction and/or fishing activities using mobile bottom-contacting gears such as beam-trawls or clam-dredges induce so far the most profound, severe and long-term effects on maerl beds^5,19–21^. Dredging causes maerl mortality both directly by breaking and burying thalli^20–22^ and indirectly through an increase in suspended material limiting light availability for photosynthetic activity and clog between branches interstices. Dredging also particularly “smoothers” maerl beds seascape and thereby reduces spatial complexity^22^. It therefore strongly modifies the composition of associated benthic fauna community with lower diversity, abundances, and biomass found in dredged compared to unfished areas^19,23,24^. Dredging-induced structural and biological changes of maerl beds are likely affecting the many important ecosystem functions displayed by this habitat in term of sediment budget, organic matter processing and therefore (“blue”) carbon storage capacity^25^. Moreover, maerl beds habitats are characterized by low resilience capacities due to: (1) the slow growth of maerl-forming algae species, and (2) the complex architecture of the beds resulting from accumulation of both live and dead maerl over periods that can exceed centuries^18^. The effect of dredging on maerl and associated benthic communities have been quite extensively demonstrated mainly based on comparisons between fished *vs* unfished areas^19,23,24^. However, only little is known regarding quantitative relationships between quantified physical disturbance intensity generated by fishing activities using bottom-contacting gears and the architecture of maerl beds, although this is key for a better assessment of the effects of physical disturbance on biodiversity-ecosystem functioning relationships in view of efficient management strategies. Assessing changes in habitat complexity and architecture of maerl beds clearly constitutes a challenging task. Up to now, aside from purely qualitative assessments^5,21^, studies focusing on beds architecture have used fastidious and time-consuming *ex-situ* measurements involving both destructive sampling and artificial rearrangements of thalli^22^. In this context, the use of Sediment Profile Imagery (SPI) techniques^26^ in maerl beds is likely to allow for a non-destructive *in-situ* acquisition of cross-section images of maerl bed and thus for the easy assessment of their vertical architecture and structural characteristics. Such an achievement would thereby facilitate *in-situ* assessments of changes in maerl beds habitat complexity across gradients.

More generally, up-scaling biodiversity-ecosystem functioning relationships and how they are impacted by disturbance to a level useful for management (generally the habitat to ecosystem scale) require an incorporation of spatial and temporal heterogeneity as well as the detection of thresholds of change in ecosystems structure and functions^27^. This is of particular importance in the framework of physical disturbance generated by fishing in the benthic compartment since these activities are known to homogenize seascapes^28^ as would do any disturbance on ecological pattern and processes^29^. It therefore highlights the need for studies assessing fine scale changes in ecosystem structure (and functions) across broad (stretched) pressure gradients in order to depict realistic view of disturbance effects on Biodiversity-Ecosystem Function relationships^30^.

Maerl beds of the Bay of Brest (a semi-enclosed bay of western France) are mainly composed by two species: *Lithothamnion corallioides* (Crouan & Crouan) and *Phymatolithon calcareum* (Adey & McKibbin)^11^. They occupy the majority of the southern part of the bay’s seafloor over shallow muddy substrata. These beds are very stable with a negligible influence of ripple marks only creating small hypsometrical differences over very long wave lengths^31^. Physical disturbance in these beds mainly results from dredge-fishing activities particularly targeting the bivalve *Venus verrucosa* (Linnaeus). The present study then aims at quantifying the impact of dredge-fishing activities on the maerl beds structure and vitality in the Bay of Brest across a fishing pressure gradient. This has been achieved through the acquisition of *in-situ* images of maerl beds cross-section using Sediment Profile Imaging system (SPI) along a high resolution dredging intensity gradient obtained through the analysis of AIS data.

## Results

### Pressure map and sampling gradient

**Figure 1:**
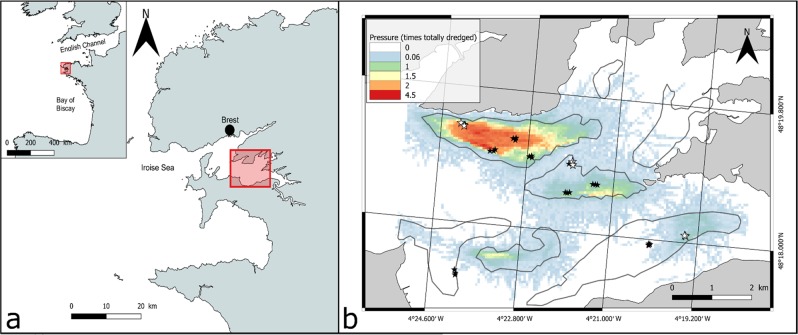
(**a**) Localisation of the Bay of Brest within the French Atlantic coast and of the study area. (**b**) Dredging intensities (Pressure) calculated over 5 years for 50 × 50 m squares within the study area together with localisation of the maerl beds (bordered with gray lines). Black stars represent stations sampled for SPI images only (black stars) and white stars the stations sampled for both SPI images and cores. Maps were created using QGIS 2.18.1 software (QGIS Development Team. QGIS Geographic Information System. Open Source Geospatial Foundation Project. http://qgis.osgeo.org).

Dredging intensity greatly varied within the study area, both among and within maerl beds (Fig. 1b). In particular, although the north-western bed was the most extensively dredged, while the one located in the southwestern as well as the two in the northeastern ends of the study area were almost free from dredging activities during the considered time window (2012–2017). Considering the 2500 m² cells units, dredging intensity ranged from 0 to 4.7 times a cell has been fully dredged during the 2012–2017 period. Image acquisition using SPI has then been performed in 30 stations located across a pressure gradient stretching from 0 to 3.26 times the cells sampled have been fully dredged (Fig. 3). These stations can be divided into 3 groups of dredging pressure with dredged area ratios ranging from 0 to 0.06, 0.61 to 1.56 and from 2.52 to 3.26, for the groups *Control*, *Moderate* and *High*, respectively (Fig. 3).

**Figure 2:**
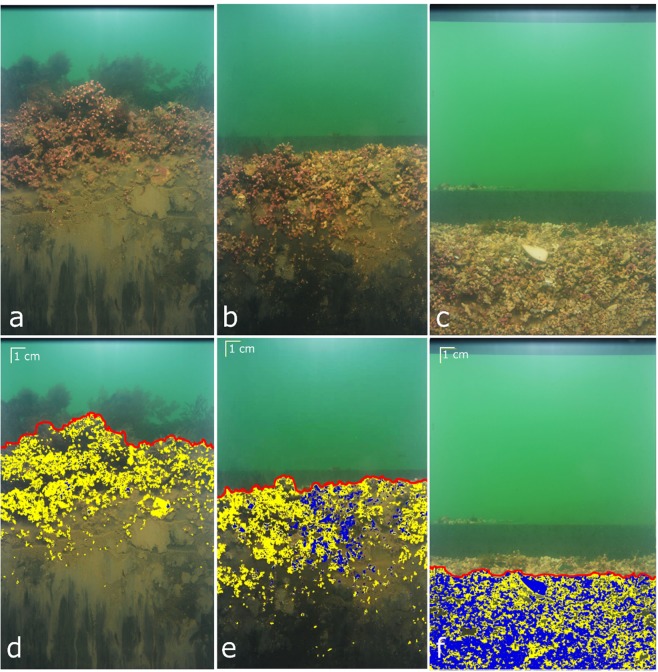
Photographs showing examples of cross-section images of maerl beds obtained with SPI in stations belonging to the Control (**a**), moderate (**b**) and High (**c**) dredging intensity groups and corresponding semi-automatically analyzed images (**d**–**f**). Red lines refer to the water-maerl interface, yellow and blue overlays to live and dead maerl, respectively.

**Figure 3:**
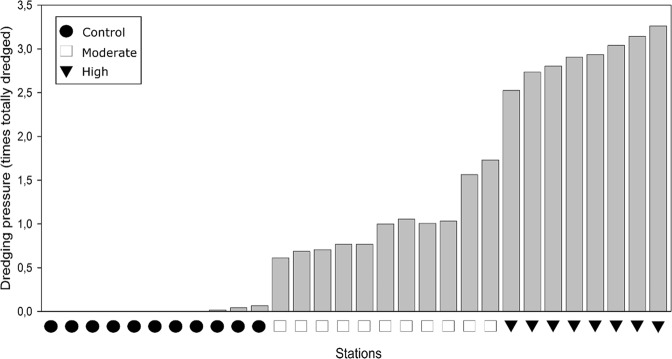
Dredging intensities (summed over 5 years) measured in the 30 sampled stations. Black circles, white squares and black triangles indicate stations belonging to the *Control*, *Moderate* and *High* dredging intensity groups, respectively.

### Effect of dredging pressure on maerl bed habitat architecture and complexity

The effect of dredging on the vertical architecture of maerl beds has been evaluated based on (1) station mean values and (2) station heterogeneity (as assessed through standard deviations) in the parameters measured from SPI images.

**Figure 4:**
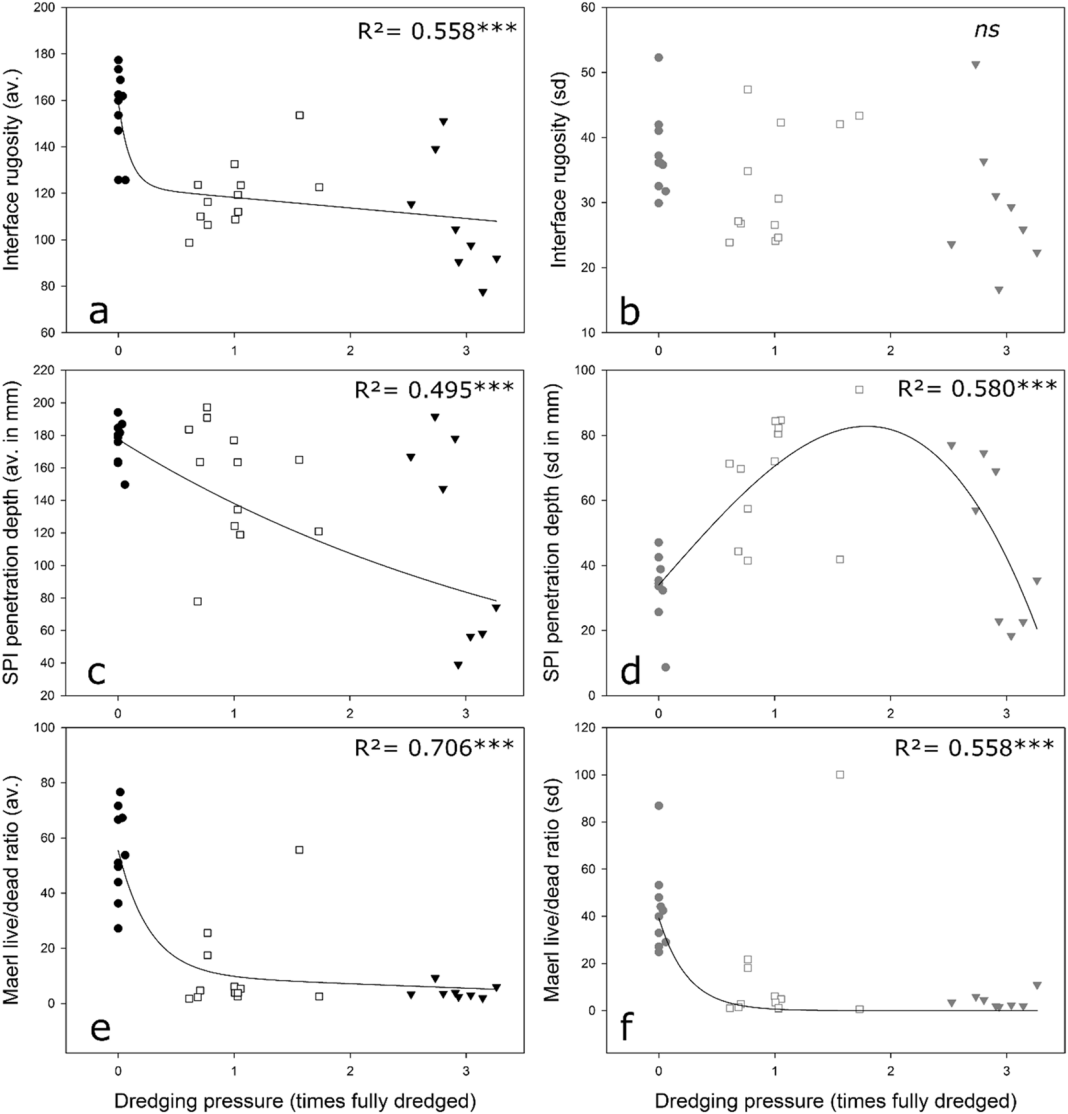
Relationships between dredging intensity and station-scale averages/standard deviations of Interface rugosity (**a**,**b**), SPI penetration depth (**c**,**d**) and maerl live/dead ratios (**e**,**f**).

**Figure 5:**
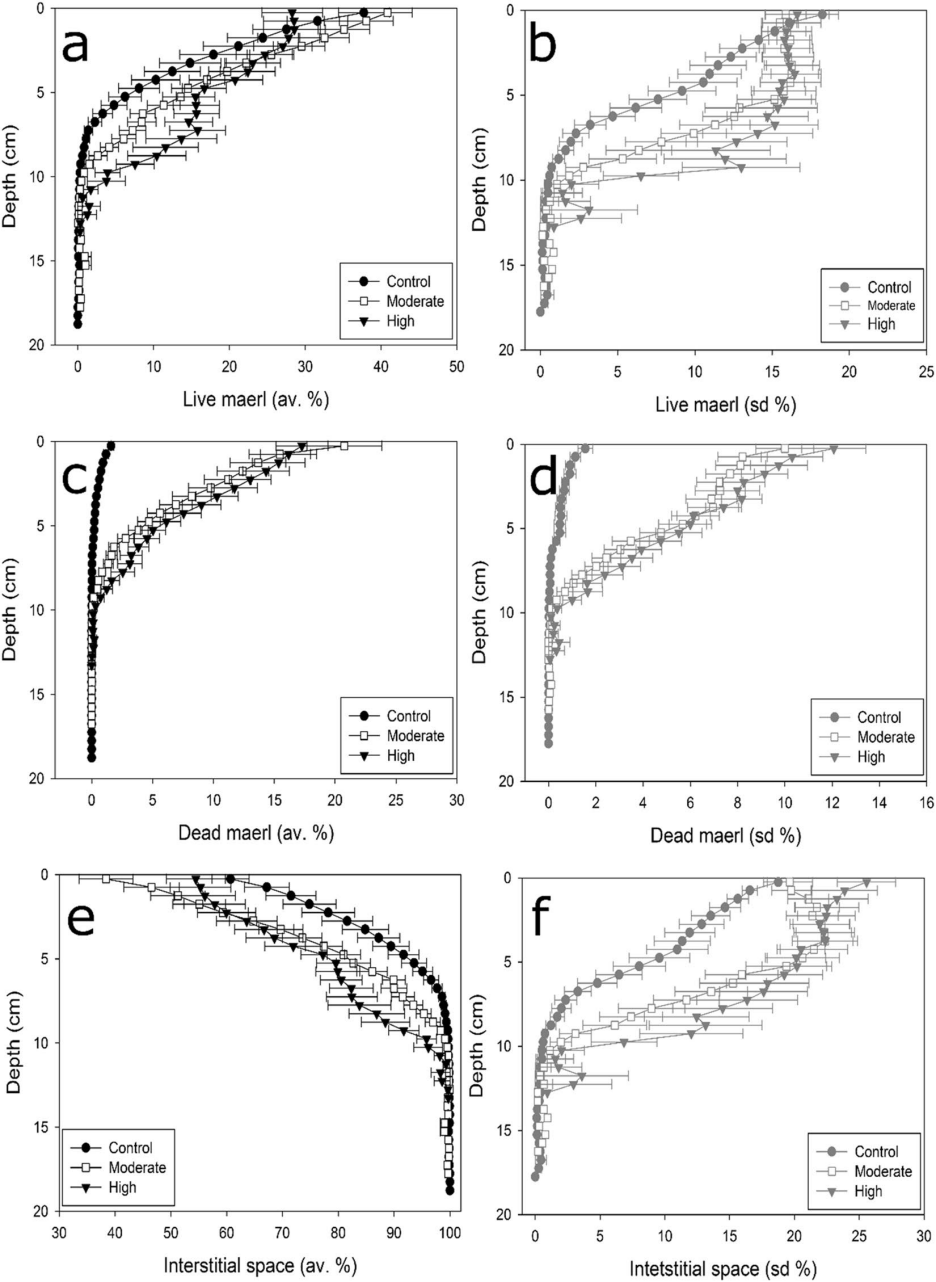
Vertical profiles of mean (±SE) station-scale averages/standard deviations of the relative proportions occupied by live maerl, dead maerl and Interstitial space in the three dredging intensity groups. Black circles, white squares and black triangles indicate Control, Moderate and High dredging intensity groups, respectively.

Overall, changes in the main univariate characteristics (interface rugosity, SPI penetration depth and maerl live/dead ratio) as well as in the vertical structure of the beds (profiles of the proportion live maerl, deal maerl, and interstitial space) all negatively correlated with dredging pressure (Figs 4, 5, Table 1). Effect(s) of dredging could also be found when looking at changes in spatial heterogeneity of these parameters, at the scales of both the station or the pressure intensity group (Figs 4, 5, Table 1). The shapes and significance of these relationships with dredging pressure however varied in function of the measured parameter and/or its nature (average or standard deviation):

**Table 1:** Results of Permanovas analyses carried out on Station-scale averages (a) and standard deviations (b).

a: Station average	Df	Main test Pseudo-F	CONTROL vs MODERATE	CONTROL vs HIGH	MODERATE vs HIGH
Live Maërl profiles	2	**3**.105*	ns	*	ns
Dead Maërl profiles	2	**15**.865***^D^	***^D^	***^D^	ns
Interstitial space profiles	2	**5**.711**	**	**	ns
Interface rugosity	2	**16**.107***	***	***	ns
SPI penetration depth	2	**38**.497***^D^	***^D^	***	ns
Maerl Live/Dead ratio	2	**37**.989***	***	***^D^	ns
**b: Station standard deviation**					
Live Maërl profiles	2	**4**.243**	*	**^D^	ns
Dead Maërl profiles	2	**18**.475**^D^	***^D^	***^D^	ns
Interstitial space profiles	2	**6**.224**^D^	**^D^	**^D^	ns
Interface rugosity	2	1.910	ns	ns	ns
SPI penetration depth	2	**10**.679**^D^	**^D^	**^D^	ns
Maerl Live/Dead ratio	2	**8**.887**	**	**^D^	ns

*, ** and *** indicate whether p(perm) values were below 0.05, 0.01 and 0.001, respectively (ns = non-significant). ^D^Indicate wheter a significant (p(perm) < 0.05) dispersion effect was detected (PERMDISP).

Average station scale interface rugosity significantly varied across the dredging intensity gradient (Fig. 4a, Table 1a) with a first exponential decay followed by slight linear decrease. Accordingly, when considering the pressure group scale, the global effect of the factor Group was highly significant with pairwise differences detected between the control group and the two others. No significant effects of dredging at the station scale (Fig. 4b, Table 1) nor group scale heterogeneities (Fig. 4a,b, Table 1) of interface rugosity were detected. Indeed, both tend to be highly variable all along the gradient.

Average SPI penetration depth significantly decreased with dredging intensity, the shape of this relationship inclining toward being linear (Fig. 4c). Such a decrease was particularly associated with lower variability in the station averages in the Control group, as indicated by the significant pairwise Permdisp dispersion tests found for comparisons of Control *vs* Moderate and Control *vs* High, respectively (Table 1). Station standard deviation of SPI penetration depth, as a proxy for spatial heterogeneity at this scale, varied across the pressure gradient following a bell-shaped curve (Fig. 4D). Significantly lower standard deviations were then found in the Control group whereas the highest ones in the Moderate group. Control groups sd SPI penetration depth were also significantly less dispersed than in the two other groups (Table 1).

Both station average and standard deviation of the Maerl live/dead ratio followed the same pattern relative to dredging intensity. This was characterized by an exponential decrease with values tending toward zero in the high intensity group (Fig. 4e,f). Dispersion of data was in the two cases also significantly lower in the High group (Table 1) whereas for example station average maerl live/dead ratio ranged from ca. 25 to 78 in the Control group and from 1 to 56 in the Moderate group.

Vertical profiles of the relative proportions of live maerl, dead maerl and interstitial spaces all varied amongst dredging pressure groups (Fig. 5, Table 1). These differences in the vertical architecture of the beds are visible while considering both station averages and standard deviations. Vertical profiles of the proportion of live maerl at station scale average and standard deviation are shown in Fig. 5a,b, respectively. Profiles measured in the stations belonging to the Control and Moderate pressure groups roughly showed the same pattern, with exponential-like decrease with depth. Proportion of live maerl however tended (no significant differences in averaged profiles, Table 1a) to be higher in the Moderate group down to a ca. 10 cm depth (Fig. 5a). They were significantly more variable at the station scale (Table 1b) with standard deviations of proportion of live maerl profiles showing,, higher and almost constant values down to a ca. 6 cm depth when compared to Control. Although live profiles in the High pressure group never significantly varied from the moderate group (Table 1a,b), both average and standard deviation profiles significantly differed from the control group (Table 1a,b) and were characterized by (1) low values at the interface with water (at 0 cm depth), and (2) value that only slowly decreased down to ca. 10 cm depth. This was particularly marked when focusing on station scale standard deviations (Fig. 5b). Vertical profiles of the proportion occupied by dead maerl clearly showed the significant differences found between the Control group compared with the other groups (Fig. 5c,d, Table 1a,b). Profiles of dead maerl were characterized by low values all along the profiles. These profiles were also significantly less variables, at both the station and the pressure group scales. Vertical profiles of the proportion of interstitial space also only significantly differed between the Control pressure group and the two others (Table 1a,b). Although the percentages of interstitial spaces were higher in the Control group all along the profiles, as seen with the profiles of the station scale averages (Fig. 5e), their station scale variability, as seen with profiles standard deviation (Fig. 5f), were lower all along the profile compared with the two other pressure groups.

**Figure 6:**
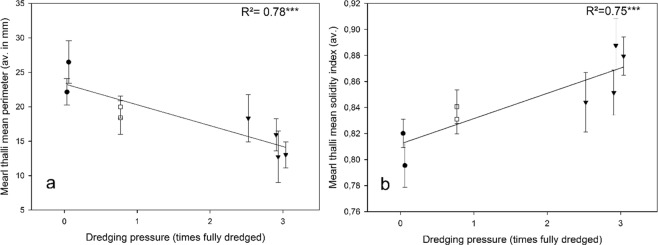
Relationships between dredging intensity and station-scale averages (+/− SD) of maerl thalli mean perimeter (**a**) and maerl thalli mean solidity index (**b**).

The two morphological indicators for maerl thalli sampled using the Plexiglas cores both significantly correlated with dredging pressure (Fig. 6). Across the dredging pressure gradient, mean perimeter, indicative of the size of the thalli, significantly decreased (Fig. 6a), while mean solidity index showed a significant increase (Fig. 6b), translating that thalli shape became less complex.

## Discussion

### Use of SPI imagery techniques to assess the impact of dredging on maerl beds

The last decades have seen an extensive development of image acquisition and analysis techniques applied to the study of marine benthos^32–34^. These technological hard- and software advancements clearly allowed for a better appreciation of *in-situ* habitat structures and complexity, from broad seascape mapping using *i*.*e*. drones or ROVs^35^, to finer scale assessment of the vertical structuration of the seabed using SPI imaging^34,36^. Due to their simplicity of acquisition, SPI images have been widely used for the assessment of benthic habitat structure and ecological quality in relation with disturbances^36–38^. In particular, they proved efficient in assessing the effect of bottom trawling on sedimentary seabeds^37,39,40^. According to such purposes, a great attention has been taken to develop common analysis conceptual framework^41^ as well as (semi)automatic image analysis tools^38^ minimizing the operator effect of the processing and thus of the information derived from so-obtained images. The deployment of SPI imagery then opens the field for a non-destructive *in-situ* access to the vertical dimension of maerl beds. SPI deployment is known to be successful in muddy soft bottoms where the device can easily penetrate into the sediment matrix^26,40^.

In the present study, the muddy nature of the sediment underlying the beds in the Bay of Brest allowed the SPI prism to penetrate enough into the matrix and therefore permitted its first use for the exploration of such biogenic habitats. Indeed, “maerly” sediments have already been SPI sampled by Smith *et al*.^40^ but (1) maerl forming algae only appeared to be a sparsely distributed habitat feature of the sediment surface among others (such as polychaetes’ tubes) and were therefore not constituting a ‘truly’ structured maerl bed, and (2) the coarse nature of the sediment underneath impeded a proper assessment of the habitat vertical structure, as noticed by these authors.

### Effect of dredging on maerl vitality

Through the analysis of *in-situ* cross-section images of maerl beds across a quantified dredging intensity gradient, we have been able to demonstrate the strong impact such activities create on a very sensitive and poorly resilient biogenic benthic habitat, in terms of both habitat forming algae vitality and bed physical architecture (habitat complexity). Although similar types of impacts have already been shown/suggested in the literature, they were mostly based on experimental dredging^20,21^ or comparisons of presumably fished *vs* presumably unfished areas^22,23,42,43^, which might complicate the interpretation of results^23,42^. Mostly these studies compared fished to unfished areas and were unable to assess the impacts of different fishing pressures on maerl bed structure. The present study conversely assessed dredging impacts on maerl beds across a quantified and continuous abrasion pressure gradient representative of the range of the dredging footprint observed within the study area (Figs 1, 2). Because of the very fine spatial scale at which both dredging intensity and maerl bed structure were assessed, this approach therefore allowed for the establishment of real world pressure/impact relationships curves (Fig. 4). It should be underlined that this kind of dataset has been identified as the only way to go forward for an accurate estimation of the effect of fishing on biogenic habitats useful for effective protection^44^. The shapes of these curves varied depending on the precise nature the relationship between the pressure and the different parameters that describe the physical structure (Interface Rugosity, Penetration depth) or vitality (Live/Dead ratio) of the maerl beds.

Maerl live/dead ratio, indicative of the vitality of the bed forming algae, clearly responded to dredging pressure according to an exponential decay tending toward 0 (Fig. 4e). It underlines that the vitality of the algae within the bed thickness is strongly affected by (physical) disturbance, even at very moderate intensities. The proportion occupied by dead maerl was significantly higher from 0 to 10 cm depth at stations belonging to the moderate and high intensity dredging groups as compared to Controls (Fig. 5c, Table 1). Along the same line, profiles also describe significantly higher proportions of live maerl down to the same depth-range at high intensities than in Controls (Fig. 5a, Table 1), indicative of the burial of live broken thalli under the action of dredges. The fact that profiles of station standard deviations of live maerl were affected on the same way, and thus already at Moderate intensities (Fig. 5b, Table 1b) would confirm this dredge-induced burial (prior to death) since at moderate intensities, there was logically less change than at high dredging intensities for the exact patch sampled using the SPI to have been only recently dredged, allowing the buried thalli to stay alive. These profiles then precise that dredging severely increased mortality through burial of broken thalli down to ca. 10 cm depth, corresponding to the length of the dredges’ teeth^45^. At high dredging pressure the beds were therefore constituted of a thick layer of dead broken pieces of maerl thalli with the noticeable presence of live thalli deeply buried and presumably soon dying off (Fig. 3c). These finding are fully coherent with Hall-Spencer and Moore^20^, who, after experimentally dredging a “pristine” maerl bed, reported a decrease in the amount of live thalli of over 70% with no sign of recovery during the 4 following years. These authors then suggested a lack of graduation in the effects of dredging on the vitality of algae that is clearly supported by our own results obtained across a quantified dredging intensity gradient. The other noticeable point regarding maerl vitality relates to the spatial extent at which the effects of dredging are measurable. Both the relationships between station average or spatial heterogeneity (measured through standard deviation) in maerl vitality proxies (live to dead ratio and dead maerl profiles) and dredging pressure suggest a binary-like response. Thereby, even when a particular sampling location (within a station) has not likely been exactly dredged, it can still be strongly affected by dredging or its consequences. This is coherent with results found in an extensive corpus of studies [e.g. 20, 21, 28, 46] showing that bottom contacting fishing gears affect the seafloor far beyond the sole footprint (i.e. ploughing track) of the gear. In particular, ploughing with dredges directly abrades the seabed with metal teeth. While a part of the so-broken (and presumably soon dead) thalli is buried, another is indeed dispersed by (tidal) currents and therefore settling in surrounding of the dredged track^21^. The same goes with fine (mud) particles suspended in large clouds subsequently blanketing the seafloor surface and clogging interstices more than 10 meters away from the ploughing track^20,21^. While the first process would spread severely damaged thalli around the dredge footprint, the second one induces algae mortality through limitation of light and oxygen availability^20^. Both processes, together with the initial physical damage produced by the dredge, contribute to an extensive decrease in maerl vitality homogeneously observed from very low dredging intensity. Interestingly, a clear outlier was detected in Fig. 4e,f which corresponded to a station where, although characterized by moderate dredging intensity, both average maerl vitality and its variability were measured as comparable to the control stations (Fig. 4e,f). A closer examination of the images taken at this particular station revealed that they were highly variable with either strongly impacted maerl beds or relatively untouched beds but characterized by thin maerl layer onto the muddy matrix. Such an observation is indeed coherent with the location of the station on the edge of a dredging footprint in the north-western bed (Fig. 1), and thus in the vicinity of the channel characterized by stronger tidal currents than in the other areas^46^. Therefore the high variability in vitality detected within the 2500 m² square could be indicative of: (1) a dredging pressure, and thereby a direct impact aggregated in the inner part (closer to the core of the bed) of the square, and (2) a negligible indirect impact at the outer edge (close to the channel) because of stronger currents, preventing the maerl layer from blanketing and allowing for the maintenance of high levels of vitality.

### Impact of dredging on maerl bed habitat architecture and complexity

In the specific context of maerl beds, SPI penetration depth is a proxy for sediment compaction, loss in fine particle and crushing of maerl thalli. Since SPI can only hardly penetrate on coarse substrata^26^ and the morphology of maerl thalli broken by dredging is comparable with gravels^22^, large penetration depths would be indicative of a well-structured and individualized maerl bed lying on pretty homogeneous mud. Whereas on the opposite small penetration depths indicate a substratum mainly constituted of fragments of broken thalli including a high proportion of dead maerl imbedded within a compacted sediment matrix (Fig. 3). This is fully coherent with the morphology analyses performed here showing thalli that, while being broken, become smaller and less complex in their shape with increasing dredging intensity (Fig. 6). Station averages of SPI penetration depth showed a close to linear decrease along the dredging intensity gradient (Fig. 4c). Although these results are also characterized by high variability, they translate a more gradual effect of dredging than the one found for maerl vitality (Fig. 4a). Conversely to the binary-like and spatially extending effect of dredging on maerl vitality that can be observed at the scale of the 2500 m² cell unit, the relationship between SPI penetration depth and dredging intensity more depended on the exact localization of the dredge ploughing track. Such an assertion is supported by the bell-shape curve representing the relationship between station-scale spatial heterogeneity in penetration depth and dredging intensity (Fig. 4d). Moderate dredging intensities indeed significantly increased spatial heterogeneity (Table 1) in creating, within the station (2500 m²), a mosaic of patches with different impacts of the dredge: heavily or lightly ploughed by the dredges themselves, scraped by the line, or simply untouched by the gears, as already noted^28,40^. Moreover, a specific practice used during dredge-fishing in the bay of Brest consists in first ploughing the sea floor during ca 2 seconds filling before lifting the dredge to be shaken for ca 5 seconds. When the dredge is emptied from finer particles, it is therefore ploughed again in the sea floor. Such a practice surely increases the heterogeneous characteristics of the direct impact at moderate dredging intensities. At higher dredging intensities, the spatial heterogeneity in SPI penetration depth clearly decreased, indicative of the fact that the whole surface of the station has been likely dredged, therefore homogenizing seascape. This is in good agreement with general assumed effect of bottom fishing gears whose main consequences consist in a smothering of seascapes^28,44,47^.

Surface rugosity from SPI images, sometimes related as (sediment-water boundary) roughness^40^, refers to the interface topography. The maintenance of such very small scale heterogeneity is at the basis for the display of important ecosystem functions^44^ such as primary production or nutrient processing^14^. Within the most common framework of use of SPI imaging techniques, *i*.*e*. in soft sediment, it can appear particularly difficult to distinguish between changes in surface rugosity values due to artificial (anthropogenic) and natural (biological) disturbances. This is particularly the case of disturbance by bottom contacting fishing gears that can either decrease or increase SPI image interface rugosity because of the potential occurrence of (1) gear-induced broken or scraped sediments, or (2) bioturbated sediments involving the presence of mounds, pits or burrows^40^. Conversely, the inherent rougher surface of maerl beds^14^ only results from the structurally complex and fragile nature of the algae thalli, pledging for a more straightforward statement that a decrease in the interface rugosity would be indicative of a destruction of the upper thalli structured layer. This was clearly confirmed by the SPI images taken during the present study (Fig. 3). Then, across the dredging intensity gradient, station scale averages of interface rugosity first showed a steep decrease from control to moderate intensities followed by a much smaller one toward high intensities (Fig. 4a). These results clearly suggest that the impact of dredging on interface rugosity appears at moderate dredging intensities, when the complex structure of the maerl bed is firstly affected. Passed that threshold, although a clear trend remained visible (Fig. 4a), it appeared difficult to attribute to the increasing dredging intensity the observed changes in interface rugosity (Table 1) as well as in station scale spatial heterogeneity (Fig. 4b, Table 1). These could however be related with the less predictable co-occurring effects of hydrodynamic since maerl beds’ structure and boundaries are known to be shaped by currents, waves and the occurrence of storms^48,49^. Broken thalli from the uppermost bed layer would thereby disseminated for further re-growing and colonization, partly explaining beds’ mobility^48,50^.

The relative lack of correlation between interface rugosity and dredging at moderate and high intensities could also be explained by the temporal aggregation of dredging chosen for the present study, *i*.*e*. a 5 years summing. Maerl beds resilience capacities are generally very low due to the very slow growth of the algae and impacts by dredging events that occurred ca. 5 years in the past still affect the habitat structure^20,45,51^, motivating the 5 years’ aggregation of dredging intensity data here. One cannot however exclude that slight increases in interface rugosity could, after several years without fishing, indicate first hint of relative restauration. Potential responsible mechanisms like (autogenic) ongoing restauration processes or recolonization by epifauna have already been recorded at comparable time-scale^23,42,52^.

### Potential consequences for ecosystem functioning

We have shown that even very moderate dredging intensities dramatically increased bed forming algae mortality, and thus at spatial scale exceeding the sole ploughing tracks of the dredges. It seems then obvious that photosynthetic activity and associated primary production supported by maerl beds^11–13^ are strongly affected as well. This of particular concern for the shallow semi-enclosed embayment such as the bay of Brest, whose functioning is severely affected by eutrophication^18^. Thereby, altering maerl beds vitality most likely also preclude their significant contributions to important ecosystem functions at the scale of the bay such as nutrient recycling and control of phytoplanktonic production^12,13^ as well as Carbon storage^11,25^. Possible loss of important ecosystem functions would also be supported by the decrease in habitat complexity we observed across the dredging intensity gradient. It appears first clearly that both decrease in the rugosity of water-maerl interface and in interstitial space associated with a compaction of the substratum are likely to reduce the exchange surface between water and maerl, and between maerl and sediment respectively, also influencing the magnitude of the carbon and solute benthic fluxes^14^. Current velocity reduction capacity of maerl beds would accordingly also be reduced with a potential impact on erosion, sedimentation rates^14^. And last but not least, both the dredging-associated drops in algae vitality and in habitat complexity likely induce, in addition to direct mortality^20^, drastic changes in the structure and diversity of the epi and in-fauna communities sheltered by maerl beds^20,21,24^ and associated functions while diminishing the number of available ecological niches and the total volume of habitat suitable for macrofauna to establish. The role of sponges in the silica cycle at the level of the whole ecosystem and for which maerl beds harbor more than 80% of the biomass is an example of the indirect consequences that maerl degradation might have on the quality of the phytoplankton in the bay^53^.

## Conclusion

The use of SPI imagery in the maerl beds in the bay of Brest allowed, within a limited timeframe, for the acquisition and analyses of a large number of samples. Coupled with very fine scale determination the intensity of dredge-fishing activities that occurred during the last 5 years, this clearly led to a consistent assessment of the slightly different impacts dredging-induced disturbances created on the different aspects of the structure of the studied maerl beds, and thus in real world conditions. Through a direct *in-situ* access to the vertical cross-section dimension of maerl beds, it also contributes to better understand consequences of physical disturbance on habitat complexity, and the potential effect on ecosystem functions supported by these fragile biogenic habitats. It therefore opens the field for future studies aiming at unravelling the relationships between (biogenic) habitat complexity, biodiversity and ecosystem functioning and how they are modified by disturbance intensity.

## Methods

### Study area

The Bay of Brest is a ca. 180 km² semi-enclosed bay located in Brittany (Western France). It is connected to the Atlantic Ocean and the Iroise Sea by a deep and narrow strait and mainly receives fresh water inputs from the Aulne and Elorn rivers (Fig. 1A). The Bay is shallow, with an average depth of ca. 8 meters and more than 50% of its surface below 5 meters depth. Sediments range from mud to coarse gravels with up to 30% of the seafloor occupied by maerl beds^54^. The present study focused on the south basin (Fig. 1B) that harbors most of the maerl of the bay. Seven different beds are identified and particularly characterized by and are subject to physical disturbance due to dredging activities generated by clam (*Venus verrucosa*) fishery that take place between October and March each year.

### Establishment of high resolution dredging pressure map

Spatiotemporal changes in the physical disturbance generated by clam-dredging activities have been assessed based on AIS (Automatic Identification System) data emitted by each fishing vessel working in the study site. This was facilitated by the strict regulation of clam-fishing in the bay that: (1) requires the fishing vessels to carry AIS transceivers, (2) only allows fishermen to daily work during a 2.5 hours’ time-slot (between 9:00 and 11:30 am) and (3) restricts the gears that can be used to a single 1.5 m wide dredge.

Briefly put, AIS recorded vessels id, position and speed at a high frequency. Vessels targeting clams were identified based on information from the The *Comité Départmental des Pêches Maritimes et des Elévages Marins du Finistère* (committee for marine fisheries and aquaculture for the Finistère department) that regulates the fishery, and typical fishing speed (when the vessel is actually dredging) for the fleet was estimated as between ca. 2.5 and 5 knots. It was therefore possible to calculate the length and position of each dredging event. These data were then aggregated within 2500 m² cells (50 m × 50 m squares) and multiplied by the width of the dredge. So-obtained dredged areas were cumulated for the 5 last fishing seasons (2012–2017), and dredging pressure was expressed in times the considered cell had been totally dredged and mapped (Fig. 1B). The choice of cumulating pressures over 5 years was motivated by the very slow growth rate of maerl algae and by their low resilience capacity to dredging^20^. Calculation was done using R programing framework (R core team), and the map produced using QGIS software (QGIS Development Team, http://qgis.osgeo.org).

### SPI acquisition

Sampling took place from the 5^th^ to the 13^th^ of April 2017. Sediment Profile Imaging system (SPI, Ocean Imaging Systems) fitted with a Nikon D7100 digital camera has been deployed from the r/v “Albert Lucas” in order to obtain *in-situ* vertical cross-section images of maerl beds (representing 14.5 × 21.8 cm) at 30 stations (Fig. 1B) located along a physical disturbance gradient defined on the basis of the above described assessment of dredging intensities. Stations were selected to encapsulate the maximum variability in calculated dredging intensity. For each station, 15 photos were taken ensuring they came from the same 2500 m² squares.

### Maerl cores sampling

In 8 stations (Fig. 1), maerl samples have been taken by scuba divers using plexiglas cores (internal diameter = 5.5 cm) between 8 and 11 cores per station were sampled. Maerl thalli were then carefully washed and dried. Thalli were then gently placed onto a mat black background and photographed using a Canon R EOS 500D digital camera (15.1 Mpixels definition) for further image analysis.

### Image analysis

The 450 so-obtained SPI images (6 M pixels resolution) were analyzed semi-automatically using a modified version of the SPIArcBase software^36^. For each image, the water-maerl interface was first drawn manually. Each pixel below this interface was therefore automatically qualified as belonging to live or dead maerl, or interstice (that can be water or sediment, indistinctly) based on its RGB color composition. The distinction between live and dead maerl has been achieved according to a unique threshold in the red channel set on the basis of good agreement between software classification and expert (JG) judgment within a selected subset of images. Examples of images obtained during the present study are shown in Fig. 2.

Interface rugosity was calculated as the sum of absolute differences in the y position of the interface for all contiguous x and divided by the width of the image^55^. Penetration depth of the SPI was measured from the average position of the interface and the bottom of the image. The surface of detected live and dead maerl were then used to calculate live/dead ratio for each image. Vertical profiles of the relative percentages of surface occupied by (1) live maerl, (2) dead maerl and (3) interstices were calculated after flattening the interface and readjusting the position of each y colmun. All profiles were assessed with a 5 mm vertical resolution.

Images of maerl thalli from cores were used in order to derive two morphological parameters, (1) the mean perimeter of thalli and (2) the mean solidity index of thalli, corresponding to the surface of a given thallus on the image plane divided by its convex surface. Solidity index then reflects the degree of complexity of maerl thalli and is comprised between 0 (fully complex shape) and 1 (no ramifications). These analyses were performed using a custom macro developed on ImageJ 1.5.2 free software^56^.

### Data analyses and statistics

All SPI measured metrics were assessed using their Station –scale averages and standard deviations, the latest as a proxy for station-scale spatial heterogeneity.

The relationships between dredging intensity and: (1) interface rugosity, (2) SPI penetration depth and (3) maerl live/dead ratio were qualified using exponential decay, exponential and linear combination, or polynomial cubic regression models. They were as well tested using the discretized grouping onto the three dredging intensity groups (*Control*, *Moderate*, *High*) using univariate Permutation Analysis of Variances (PERMANOVAS) and associated dispersion tests (PERMDISP, 57) on untransformed data. Euclidean distances were used and the design consisted in a single fixed factor *‘Dredging intensity’* (3 levels: *Control*, *Moderate*, *High*). Pairwise tests were performed as well to distinguish differences between groups.

Differences in vertical profiles of the relative proportions occupied by (1) live, (2) dead and (3) interstitial space amongst the three groups were tested using (multivariate) PERMANOVAS and associated dispersion tests (PERMDISP) carried out on normalized data from the 0–10 cm depth and using the same distance and design as for univariate tests.

Additionally, relationships between dredging intensity and maerl thalli (1) mean perimeter and (2) mean solidity index ratio were qualified using linear models. However, they were not analyzed for the discretized pressure groups because of the low number of stations.

All statistical analyses were performed using PERMANOVA + add-on for PRIMER 7 software^57^.

## References

[1] Lemoine P (1910). Répartition et mode de vie du maërl (Lithothamnium calcareum) aux environs de Concarneau (Finistère). Annales de l’Institut Océanographique, Paris.

[2] Grall J, Le Loc’h F, Guyonnet B, Riera P (2006). Community structure and food web based on stable isotopes (delta15N and delta 13C) analysis of a North Eastern Atlantic maerl bed. J. Exp. Mar. Bio. Ecol..

[3] Foster MS, Amado Filho GM, Kamenos N, Riosmena-Rodriguez R, Steller DL (2013). Rhodoliths and rhodolith beds. Smithson. Contrib. Mar. Sci..

[4] Hall-Spencer JM, White N, Gillespie E, Gillham K, Foggo A (2006). Impact of fish farms on maerl beds in strongly tidal areas. Mar. Ecol. Prog. Ser..

[5] Barberá C (2003). Conservation and management of northeast Atlantic and Mediterranean maerl beds. Aquat. Conserv. Mar. Freshw. Ecosyst..

[6] Hall-Spencer JM (1998). Conservation issues relating to maerl beds as habitats for molluscs. Journal of Conchology Special Publication.

[7] Sciberras M (2009). Habitat structure and biological characteristics of a maerl bed off the northeastern coast of the Maltese Islands (central Mediterranean). Mar. Biodivers..

[8] Neill KF, Nelson WA, D’Archino R, Leduc D, Farr TJ (2015). Northern New Zealand rhodoliths: assessing faunal and floral diversity in physically contrasting beds. Mar. Biodivers..

[9] Peña V, Bárbara I, Grall J, Maggs CA, Hall-Spencer JM (2014). The diversity of seaweeds on maerl in the NE Atlantic. Mar. Biodivers..

[10] Kamenos N, Moore P, Hall-Spencer JM (2004). Nursery-area function of maerl grounds for juvenile queen scallops Aequipecten opercularis and other invertebrates. Mar. Ecol.Prog. Ser..

[11] Martin S (2005). Comparison of Zostera marina and maerl community metabolism. Aquat. Bot..

[12] Martin S, Clavier J, Chauvaud L, Thouzeau G (2007). Community metabolism in temperate maerl beds. I. Carbon and carbonate fluxes. Mar. Ecol. Prog. Ser..

[13] Martin S, Clavier J, Chauvaud L, Thouzeau G (2007). Community metabolism in temperate maerl beds. II. Nutrient fluxes. Mar. Ecol. Prog. Ser..

[14] Attard KM (2015). Benthic oxygen exchange in a live coralline algal bed and an adjacent sandy habitat: an eddy covariance study. Mar. Ecol. Prog. Ser..

[15] Riosmena-Rodriguez R., Nelson W. & Aguirre, J. (eds). *Rhodolith/maërl beds: a global perspective*. Coastal Research Library 15, Springer International Publishing Switzerland, 368 pp (2017).

[16] Amado-Filho GM (2012). Rhodolith Beds Are Major CaCO_3_ Bio-Factories in the Tropical South West Atlantic. PLoS One..

[17] Martin, S. & Hall-Spencer, J. M. Effects of Ocean Warming and Acidification on Rhodolith/Maër Beds. In R. Riosmena-Rodríguez *et al*. (eds), *Rhodolith/Maërl Beds: A Global Perspective*, Coastal Research Library 15, Springer International Publishing Switzerland, 368 pp (2017).

[18] Grall J, Hall-Spencer JM (2003). Problems facing maerl convervation in brittany. Aquatic Conserv: Mar. Freshw. Ecosyst..

[19] De Grave S, Whitaker A (1999). Benthic Community Re-adjustment following Dredging of a Muddy-Maerl Matrix. Mar. Pollut. Bull..

[20] Hall-Spencer JM, Moore P (2000). Scallop dredging has profound, long-term impacts on maerl habitats. ICES J. Mar. Sci..

[21] Hauton C, Hall-Spencern JM, Moore P (2003). An experimental study of the ecological impacts of hydraulic bivalve dredging on maerl. ICES J. Mar. Sci..

[22] Kamenos N, Moore P, Hall-Spencer JM (2003). Substratum heterogeneity of dredged vs un-dredged maerl grounds. J. Mar. Biol. Assoc. United Kingdom..

[23] Cabanellas-Reboredo M (2017). Morpho-demographic traits of two maërl-forming algae in beds with different depths and fishing histories. Aquat. Conserv. Mar. Freshw. Ecosyst..

[24] Coquereau L, Lossent J, Grall J, Chauvaud L (2017). Marine soundscape shaped by fishing activity. R. Soc. Open Sci..

[25] van der Heijden LH, Kamenos NA (2015). Reviews and syntheses: Calculating the global contribution of coralline algae to total carbon burial. Biogeosciences.

[26] Germano, J., Rhoads, D., Valente R., Carey, D. & Solan, M. The use of sediment profile imaging (SPI) for environmental impact assessments and monitoring studies: lessons learned from the past four decades. In Oceanography and Marine Biology: An Annual Review, Vol. 49, eds R. Gibson, R. J. Atkinson, J. D. Gordon, pp. 235–98 (2011).

[27] Hewitt JE, Thrush SF, Dayton PD (2008). Habitat variation, species diversity and eclogical functioning in a marine system. J. Exp. Mar. Biol. Ecol..

[28] Thrush SF (2002). & Dayton, P. Disturbance to marine benthic habitats by trawling and dredging: implications for marine biodiversity. Annu. Rev. Ecol. Sys..

[29] Fraterrigo JM, Rusak JA (2008). Disturbance-driven changes in the variability of ecological patterns and processes. Ecol. Let..

[30] Snelgrove PVR, Thrush SF, Wall DH, Norkko A (2014). Real world biodiversity–ecosystem functioning: a seafloor perspective. Trends Ecol. Evol..

[31] Berthois L, Guilcher A (1959). Les bancs de Saint Marc et du Moulin Blanc (rade de Brest) et remarques sur la sédimentationdu Maërl (Lithothamnion calcareum). Cahiers Océanographies du C.O.E.C. XI.

[32] Smith CJ, Banks AC, Papadopoulou KN (2007). Improving the quantitative estimation of trawling impacts from sidescan-sonar and underwater-video imagery. ICES J. Mar. Sci..

[33] Stokes MD, Deane GB (2009). Automated processing of coral reef benthic images. Limnol. Oceanogr. Methods.

[34] Romero-ramirez A (2016). Development and validation of a video analysis software for marine benthic applications. J. Mar. Syst..

[35] Mérillet L (2018). Using underwater video to assess megabenthic community vulnerability in the grande Vasière (Bay of Biscay). Environmental Conservation.

[36] Nilsson HC, Rosenberg R (2000). Succession in marine benthic habitats and fauna in response to oxygen deficiency: analysed by sediment profile-imaging and by grab samples. Mar. Ecol. Prog. Ser..

[37] Rosenberg R, Nilsson HC, Grémare A, Amouroux JM (2003). Effects of demersal trawling on marine sedimentary habitats analysed by sediment profile imagery. J. Exp. Mar. Biol. Ecol..

[38] Romero-Ramirez A, Grémare A, Desmalades M, Duchêne JC (2013). Semi-automatic analysis and interpretation of sediment profile images. Environ. Model. Softw..

[39] Nilsson HC, Rosenberg R (2003). Effects on marine sedimentary habitats of experimental trawling analysed by sediment profile imagery. J. Exp. Mar. Biol. Ecol..

[40] Smith CJ, Rumohr H, Karakassis I, Papadopoulou KN (2003). Analysing the impact of bottom trawls on sedimentary seabed with sediment profile imagery. J. Exp. Mar. Biol. Ecol..

[41] Nilsson HC, Rosenberg R (1997). Benthic habitat quality assessment of an oxygen stressed fjord by surface and sediment profile images. J. Mar. Syst..

[42] Barberá C (2017). Maerl beds inside and outside a 25-year-old no-take area. Mar. Ecol. Prog. Ser..

[43] Ordines F (2017). Why long term trawled red algae beds off Balearic Islands (western Mediterranean) still persist?. Reg. Stud. Mar. Sci..

[44] Kaiser MJ, Collie JS, Hall SJ, Jennings S, Poiner IR (2002). Modification of marine habitats by trawling activities: prognosis and solutions. Fish. Fish..

[45] Hall-Spencer JM, Grall J, Moore PG, Atkinson RJA (2003). Bivalve fishing and maerl-bed conservation in France and the UK-retrospect and prospect. Aquatic Conserv: Mar. Freshw. Ecosyst..

[46] Gregoire G, Ehrhold A, Le Roy P, Jouet G, Garlan T (2016). Modern morpho-sedimentological patterns in a tide-dominated estuary system: the Bay of Brest (west Britanny, France). J. Maps.

[47] Jones JB (1992). Environmental impact of trawling on the seabed: a review. New Zeal. J. Mar. Fresh..

[48] Dutertre M, Grall J, Ehrhold A, Hamon D (2015). Environmental factors affecting maerl bed structure in Brittany (France). Eur. J. of Phycol..

[49] Joshi S, Duffy GP, Brown C (2017). Mobility of maerl-siliclastic mixtures: Impact of waves, currents and events. Est. Coast. Shelf. Sci..

[50] Scoffin TP, Stoddart DR, Tudhope AW, Woodroffe C (1985). Rhodoliths and coralliths of Muri Lagoon, Rarotonga, Cook Islands. Coral Reefs.

[51] Collie JS, Hall SJ, Kaiser MJ, Poiner IR (2000). A quatitative analysis of fishing impacts on shelf-sea benthos. Journal of Animal Ecology.

[52] Sheehan EV, Bridger D, Cousens SL, Attrill MJ (2015). Testing the resilience of dead maerl infaunal assemblages to the experimental removal and re-lay of habitat. Mar. Ecol. Prog. Ser..

[53] Lopez-Acosta M, Leynaert A, Grall J, Maldonado M (2018). Silicon consumption kinetics by marine sponges: An assessment of their role at the ecosystem level. Limnol. Oceanogr..

[54] Grall, J. Biodiversité spécifique et fonctionnelle du maërl: réponses à la variabilité de l’environnement côtier. Phd thesis. Université de Bretagne Occidentale, Brest (2002).

[55] Friedman A, Pizarro O, Williams SB, Johnson-Roberson M (2012). Multi-Scale Measures of Rugosity, Slope and Aspect from Benthic Stereo Image Reconstructions. PLoS One..

[56] Schneider CA, Rasband WS, Eliceiri KW (2012). NIH Image to ImageJ: 25 years of image analysis”. Nature methods.

[57] Anderson, M. J., Gorley, R. N. & Clarke, K. R. PERMANOVA+ for PRIMER: guide to software and statistical methods. PRIMER-E, Plymouth (2008).

